# “If I had known, I would have applied”: poor communication, job dissatisfaction, and attrition of rural health workers in Sierra Leone

**DOI:** 10.1186/s12960-018-0311-y

**Published:** 2018-09-24

**Authors:** Vijay Narayan, Grace John-Stewart, George Gage, Gabrielle O’Malley

**Affiliations:** 1 Department of Global Health, University of Washington, Seattle, United States of America; 2 College of Medicine and Applied Health Sciences, University of Sierra Leone, Freetown, Sierra Leone

**Keywords:** Sierra Leone, Health workforce retention, Distribution, Job satisfaction, Communication, Policies, Employee benefits, Healthcare worker, Motivation, Rural and remote areas

## Abstract

**Background:**

Sierra Leone’s health outcomes rank among the worst in the world. A major challenge is the shortage of primary healthcare workers (HCWs) in rural areas due to especially high rates of attrition. This study was undertaken to determine the drivers of job dissatisfaction and poor retention among Sierra Leone’s rural HCWs.

**Methods:**

Interviews were conducted with 58 rural and 32 urban primary HCWs in Sierra Leone’s public health sector, complemented by key informant discussions and review of national policy documents. HCW interviews included (1) semi-structured discussion, (2) questionnaire, (3) card sort about HCW priorities, and (4) free-listing of most pressing challenges and needs. Sampling for HCW interviews was stratified purposive, emphasizing rural HCWs.

**Results:**

Among 90 HCWs interviewed, 67% were dissatisfied with their jobs (71% rural vs 52% urban) and 61% intended to leave their post (75% rural vs 38% urban). While working and living conditions and remuneration were significant factors, a major reason for rural HCW disenchantment was their inability to access worker rights, benefits, and advancement opportunities. This was caused by HCWs’ lack of knowledge about human resource (HR) policies and procedures, as well as ambiguity in many policies and inequitable implementation. HCWs reported feeling neglected and marginalized and perceived a lack of transparency. These issues can be attributed to the absence of systems for regular two-way communication between the Ministry of Health and HCWs; lack of official national documents with up-to-date, clear HR policies and procedures for HCWs; pay statements that do not provide a breakdown of financial allowances and withholdings; and lack of HCW induction.

**Conclusions:**

HCWs in Sierra Leone lacked accurate information about entitlements, policies, and procedures, and this was a driver of rural HCW job dissatisfaction and attrition. System-oriented, low-cost initiatives can address these underlying structural causes in Sierra Leone. These issues likely apply to other countries facing HCW retention challenges and should be considered in development of global HCW retention strategies.

**Electronic supplementary material:**

The online version of this article (10.1186/s12960-018-0311-y) contains supplementary material, which is available to authorized users.

## Backround

The severe shortage of healthcare workers (HCWs) in rural communities of low-income countries is a global crisis, driving poor health outcomes by reducing access to medical care and quality of health service delivery [1–4]. In the many sub-Saharan African countries with majority rural populations, poor retention of HCWs in rural areas is especially problematic [5, 6].

The situation is especially stark in Sierra Leone, whose health indices rank among the very worst in the world in terms of its maternal, infant, and under-5 mortality rates, as well as overall life expectancy [7, 8]. Devastating Ebola and cholera epidemics in recent years have exacerbated Sierra Leone’s health crisis [9, 10].

Despite 63% of Sierra Leone’s population residing in rural areas, only 33% of the country’s health professionals work at rural health posts [11, 12]. Many HCWs in Sierra Leone refuse their rural postings, and attrition rates among rural HCWs are high [13, 14]. At the same time, government initiatives—such as the Free Healthcare Initiative and removal of user fees for malaria and tuberculosis care—have increased patient demand [15]. With insufficient numbers of well-equipped and motivated HCWs in rural areas, Sierra Leone’s health system cannot meet the essential health needs of the majority rural population. Adding to this challenge, the country has very few medical doctors (only 0.3 per 10 000 population), and the few doctors work exclusively in urban or peri-urban areas [16, 17]. The population largely depends on the government for health services: public health facilities provide 70% of the services in the country, while private and mission facilities provide the remaining 30% [12]. See Additional file 1 for an overview of Sierra Leone’s health system.

Reasons for rural attrition and mal-distribution in low-income countries have been identified in numerous studies and reviews. Commonly cited factors affecting rural job dissatisfaction include working and living conditions, salary and financial allowances, access to career development opportunities and promotion pathways, supervision and management at the health facility, support systems, and family considerations [18–24]. According to research by Wurie et al., many of these factors contribute to job dissatisfaction among Sierra Leone’s rural health workers [25].

What has not been well documented, however, is how lack of HCW knowledge about human resource (HR) policies/procedures and poor communication between the government and its HCWs negatively impacts HCW retention in rural areas. This study examines how these knowledge and communication dynamics contribute to the demotivation of rural health workers and proposes recommendations for strengthening HCW capacity to navigate the human resources for health system of which they are an essential part.

## Methods

### Study design

**Fig. 1:**
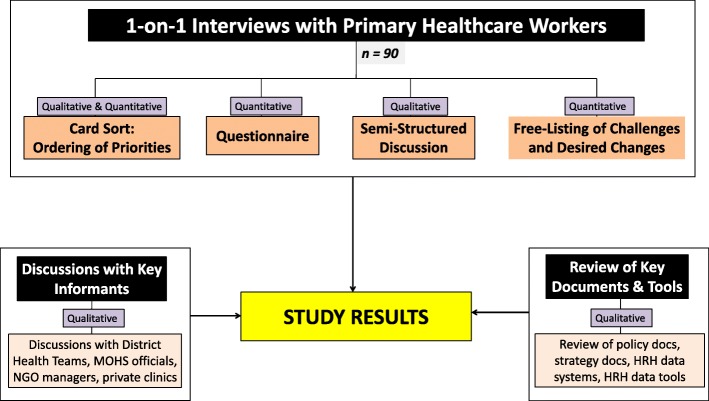
Design of health workforce retention study. Abbrev: MOHS = Ministry of Health and Sanitation, HRH = Human Resources for Health

Data for this mixed-method cross-sectional study were collected in Sierra Leone in 2014–2015. Data were collected via in-depth interviews with primary HCWs at public-sector health facilities throughout the country (*N* = 90), key informant discussions with governmental and non-government stakeholders (*N* = 37), and a review of policy documents and electronic data tools (Fig. 1). Data collection took place prior to Sierra Leone’s Ebola outbreak.

Key informant discussions were held with district and national government health officials, managers from non-governmental organizations (NGOs), staff from mission and private health facilities, and other relevant stakeholders. Discussions sought to shed light on systemic gaps undermining health workforce retention.

HCW interviews elicited qualitative and quantitative data about their individual professional and personal experiences, challenges, factors influencing their intentions to stay or leave a health posting, and types of changes that would encourage them to stay at their current posts. Interviews included four components: a 30-question survey adapted from previous studies conducted in Uganda and Kenya [26], a semi-structured discussion in which the health workers described their personal experiences, a listing exercise where health workers were asked to free-list in order of priority a maximum of seven of their most important workplace challenges and seven things they would want the government to do to increase their desire to stay at their current posting, and a card sort activity in which health care workers were asked to rank-order 13 cards depicting factors affecting job satisfaction frequently referenced in the general literature. An additional file lists the 13 factors in the card sort and describes the method (see Additional file 2).

Interviews were conducted at the health facility in a private place in the language that was most comfortable to the HCW (English, Krio, or both). No personal identifiers were documented, and written consent was obtained before the interview. Interviews lasted approximately 1.5 h and were audio-recorded. The card sort, free lists, and survey responses were documented on a data collection tool.

The document review provided additional context to the findings from the HCW interviews. Reviewed documents included the Civil Service Code of Rules and Regulations, Human Resources for Health Policy, Human Resources for Health (HRH) Strategic Plan, Essential Healthcare Package, Primary Healthcare Handbook, and National Health Training Plan. The review also included the electronic and paper tools used to collect and manage HRH data at the district and national levels.

### Sampling and participant selection

We used maximum variation sampling and purposively sampled individuals across five strata, including type of setting, health facility level, health worker cadre, geographical region and district, and supervisory status (Table 1). Facilities were selected with the help of district health management teams (DHMT), and in each district, study investigators prioritized the most “hard-to-reach” health facilities, while attaining a balance of cadres and facility levels. Rural health facilities were oversampled because the study’s primary focus was the rural workforce. The study achieved regional balance, including all 13 districts and 6–8 chiefdoms within each district. Health worker interviews included all cadres involved in primary healthcare at public-sector health facilities (state-registered nurse (SRN), state-enrolled community health nurse (SECHN), community health officer (CHO), community health assistant (CHA), maternal child health aide (MCHA)).

**Table 1:** Sampling strata for healthcare worker interviews

Purposive sampling: strata	
Type of setting	
Urban	
Peri-urban	
Rural	
Supervisory status	
Supervisor	
Non-supervisor	
Facility level	
Hospital	
Community health center (CHC)	
Community health post (CHP)	
Maternal-child health post (MCHP)	
Cadre	
State-registered nurse (SRN)	
State-enrolled community health nurse (SECHN)	
Community health officer (CHO)	
Community health assistant (CHA)	
Maternal child health aide (MCHA)	
Region and district	
All 4 regions (North, South, East, West)	
All 13 districts (Western Area, Pujehun, Bonthe, Kono, Kailahun, Kenema, Bo, Bombali, Port Loko, Kambia, Koinadugu, Tonkolili)	

Only one HCW was interviewed at any single rural facility, and a maximum of two HCWs were interviewed at any hospital. Upon reaching each facility, interviewers explained the study to the site supervisor and the chosen HCW, who was then asked if he/she wanted to participate. No HCW refused to participate in the study.

Convenience sampling was used for selection of key informants. For the review of key documents and tools, sampling was inclusive of all accessible national documents that were relevant to the health workforce and delivery of primary healthcare services.

Given our sampling method and sample size, our study findings are not generalizable to the entire health workforce. Rather, our intention was to provide an in-depth examination and description of dissatisfaction and demotivation among a purposive sample of health care workers so as to better inform policies for improving health workforce retention in Sierra Leone.

### Data analysis

Audio recordings for the health worker interviews were translated into English where necessary and transcribed. Interview transcripts, national documents, and photos taken at health facilities were imported and managed in Atlas.Ti. We used thematic analysis to generate a nuanced description of health worker perspectives and experiences within the context of facility infrastructure and national policies [27, 28]. Inductive analysis was used to develop primary and axial codes which formed an initial code book. Codes were then modified and refined through “test-coding” of several transcripts. The resulting final codebook was then used with all transcripts. Coded text and images from all data sources were analyzed and synthesized to identify key themes in answer to the research questions.

Preliminary results were shared with co-researchers in country, and their feedback was incorporated into the analysis. Quantitative data from card sorts, free-list rankings of challenges and desired interventions, and questionnaires were analyzed using Microsoft Excel and SPSS. The data analyses from the different methods were synthesized and triangulated [29].

### Ethical considerations

Ethical approval for the study was obtained from the Sierra Leone Research and Ethics Committee, as well as the University of Washington Institutional Review Board.

## Results

Out of 90 interviewed HCWs, 58 were working in rural facilities and 32 were in urban or peri-urban facilities (Table 2).

**Table 2:** Sample strata breakdown and study participant characteristics

Sample strata breakdown		
Type of setting		
Rural facilities	58	64%
Urban and peri-urban facilities	32	36%
Total	90	
Health facility level		
Hospital	29	32%
Community health center (CHC)	27	30%
Community health post (CHP)	20	22%
MCH post (MCHP)	14	16%
Total	90	
Cadre of health worker		
SRN (state-registered nurse)	14	16%
SECHN (state-enrolled community nurse)	25	28%
CHO (community health officer)	16	18%
CHA (community health assistant)	10	11%
MCHA (maternal-child health aide)	25	28%
Total	90	
Region of health worker		
North	28	31%
South	25	28%
East	23	26%
West (Freetown)	14	16%
Total	90	
Participant characteristics		
Age of health workers		
Mean age		40.1 years
Median age		41.0 years
Gender of health workers		
Female	57	37%
Male	33	63%
Total	90	
Region of origin		
Working in region of birth	50	56%
Working in another region	40	44%
Total	90	
Professional work experience		
0–2 years	5	5%
2–5 years	27	30%
5–10 years	25	28%
11–15 years	14	16%
> 15 years	19	21%
Total	90	
Mean # years working (total)		9.9 years
Mean # years at current post		2.7 years

**Fig. 2:**
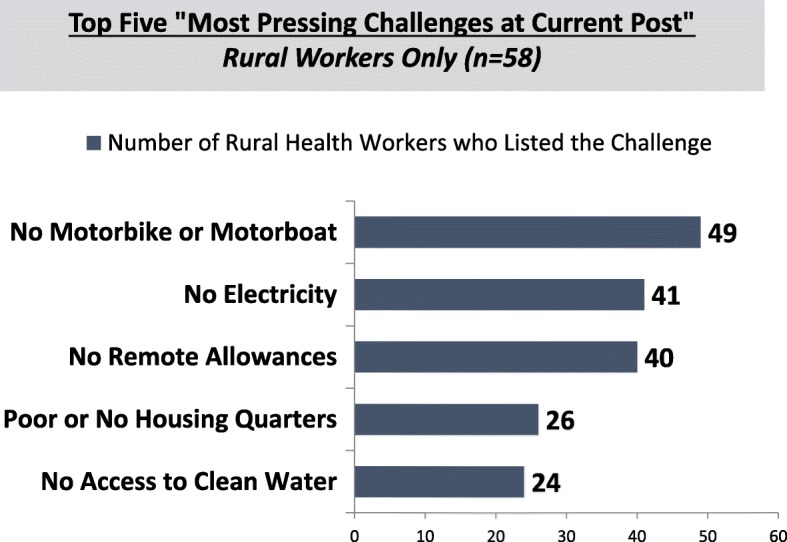
Top five “most pressing challenges at current post” listed by rural health workers (*n* = 58)

Of 90 interviewed HCWs, 61% reported that they intended to leave their current posting within 1 year. Rates of “intention to leave” were higher in rural than urban workers (75% vs 38%, respectively). Rural HCWs reported lower job satisfaction than urban HCWs (2.61 vs. 3.31 on a 5-point Likert scale). Substandard living/working conditions and remuneration gaps were significant factors affecting intention to leave among rural HCWs. Most rural HCWs in this study were living and working with inadequate or no housing, electricity, clean water, and means for basic transport, while simultaneously lacking essential equipment needed to do their jobs effectively. Many rural HCWs were not receiving their salaries or financial allowances. Figure 2 shows the top five most pressing challenges affecting job satisfaction free-listed by the interviewed rural HCWs (*n* = 58).

Among interviewed urban and peri-urban HCWs, the most pressing challenges reported were lack of housing quarters, inadequate medical supplies and stock out of medicines at health facilities, low salary, and lack of career advancement opportunities.

However, beyond the lack of financial and material support, we found a major underlying reason for HCW disenchantment was HCWs’ lack of knowledge and information about their entitlements as civil servants, national human resource policies (e.g., eligibility criteria for career advancement opportunities), and proper procedures for obtaining benefits (such as annual leave or medical care). This was compounded by the ambiguity of many HR policies pertaining to HCW opportunities and rights. The information and communication gaps disproportionately affected rural HCWs due to their isolation and distance from the central government. However, despite greater proximity and access to the regional or central government, urban HCWs were also detrimentally affected since they were often getting unreliable or wrong information.

### Lack of knowledge has prevented health workers from accessing their employee rights

Lack of accurate knowledge about policies and procedures prevented many HCWs from benefitting from career advancement opportunities, being absorbed onto the national payroll or being promoted, receiving paid annual leave, going to the right people/places in the government for resolution of professional problems, and so on. HCWs lacked awareness of policy and programming changes at the national level that affected their well-being, such as the shift to a new promotion system (Scheme of Service). As one rural HCW commented,

They don’t give us any information, so how are we to plan our lives and get the government opportunities? We don’t know what to do to get this opportunity, or who to go to for fixing this problem. I don’t know why they made this or that decision. (SECHN 34)

HCWs are entitled to paid annual vacation leave of 1 month. However, as shown in Table 3, only 43% of the interviewed HCWs knew about this policy, only 29% knew the steps to apply for it, and consequently only 16% reported ever receiving an annual leave allowance.

Further, only 2% of upcountry workers benefitted from the allowance, compared to 93% of Freetown workers. According to a rural HCW,

I never even applied for [annual] leave because my family is here and I have nowhere else I would go. But if I had known about the extra month of salary, I would have applied every year. We need that. We have earned that allowance. (SECHN-Midwife 75)

**Table 3:** Health worker knowledge and utilization of “annual leave” entitlement

Knows about annual leave and that it includes financial “allowance” (extra month of salary)		39 out of 90	43%
	Freetown city (western region)	14 out of 14	100%
	12 upcountry districts (north, south, and east regions)	25 out of 76	33%
Knows process for applying for annual leave		26 out of 90	29%
	Freetown city (western region)	13 out of 14	93%
	12 upcountry districts (north, south, and east regions)	13 out of 76	16%
Received “leave allowance”		14 out of 90	16%
	Freetown city (western region)	13 out of 14	93%
	12 upcountry districts (north, south, and east regions)	1 out of 76	2%

### Absence of accurate information fuels conjecture, speculation, and disenchantment

When HCWs lack information, they often make assumptions which are inaccurate. Uninformed conjecture can lead to disenchantment with the government that may be unwarranted. For example, prior to 2013, the government had a provision called “Study Leave with Pay” which enables HCWs to advance their careers by continuing to pay them while they attend school. In 2013, the government stopped the “Study Leave with Pay” provision. However, the information about halting the policy and the rationale was not effectively communicated to HCWs. Only 9% of HCWs (8/90) knew about the policy change. One rural MCH aide had heard from colleagues that only the MCH aides were being denied career advancement opportunities. Since the MCH aide had not received any official information, she and her colleagues interpreted the measure as marginalization of their entire cadre:

Every day, the government sends CHOs, SECHNs, midwives, and other cadres to study. The government never thinks of that for us- we the MCH Aides. I don’t know why they don’t want us to advance. The government doesn’t respect us, even when we do so much of the work. (MCH Aide 27)

The MCH aide was unhappy that she and her colleagues were being ignored as compared to other cadres. This belief, which stemmed from lack of knowledge and inaccurate assumptions, was the primary reason given by the interviewed HCW for her overall job dissatisfaction.

### Inability to know the breakdown of “composite salary” has disempowered HCWs

HCWs only see the total amount of money placed in their bank account every month. They do not receive a statement showing if and what amounts of money they are receiving for housing, medical risk, and transport, as well as what is withheld for social security and taxes. A rural HCW with no housing quarters expressed unhappiness that the government was not providing additional funds to compensate for his high rent costs. Since he did not have a disaggregated pay statement, he did not know if the issue to redress was (1) the lack of housing allowance, or (2) the housing allowance is provided but too small, or (3) the housing allowance is sufficient but the “base salary” is too small. He explained this challenge:

I have never seen my pay breakdown. They just send whole salary amount into my account. How can I know if I get housing allowance? I don’t even know ‘this is for what’, or ‘that is for what’. That info has never been given to anybody. So I don’t know which [thing] is the problem. (CHO 02)

“Remote station allowances” were supposed to be provided as an incentive to HCWs to work in rural areas. Many interviewed HCWs reported not receiving these allowances for at least the previous 1 year. However, officials in several districts said the HCWs were receiving the allowances but had no way of knowing because their pay statements were not disaggregated. National-level officials later confirmed that the remote allowances had indeed stopped. This example shows how aggregated pay statements have been so problematic: HCWs had no recourse because they were told that they were receiving the remote allowances, but could not access the information which would have enabled them to confirm or refute that assertion.

### Lack of clarity in policies has led to confusion and inconsistent implementation

In numerous areas pertaining to HCW benefits, there was no clear policy from the Ministry of Health and Sanitation (MOHS) HRH Directorate or Human Resource Management Office (HRMO). In other cases, there was an official policy position at high levels in the MOHS, but it had not been disseminated to the key national or district officials involved in implementation; this was especially the case when it involved changes to existing policies and programs.

We found confusion and inconsistency among health workers, MOHS and district health officials, HRMO staff, and other relevant stakeholders in the policy for provision of medical care for HCWs and their families, the eligibility criteria for “study leave with pay” and current status of that benefit, the status of remote allowances and which health workers were eligible, and the exact procedures HCWs should follow for absorption onto payroll and salary promotion, among many others. The only reference document for these policies was the Civil Service Code [30], which articulates the policies and entitlements for civil servants in Sierra Leone. However, few HCWs were aware of the Civil Service Code, and even fewer HCWs had obtained a copy of the Civil Service Code since its printing expense made it hard to access.

While an important document for HCWs to have, many of the Civil Service Code’s provisions are non-specific or ambiguous, and outdated since the Code was created 10 years earlier and meant to be broad so it could apply to all civil servants for an extended time period. For example, one provision in the Code reads that “civil servants shall receive a medical care allowance to be determined from time to time.” Based on this wording, HCWs do not know if they are supposed to be provided with this allowance at the current time, and in what amount. The Civil Service Code also does not reflect programming changes such as the switch to a different performance appraisal system for MOHS staff, or changes in implementation of a policy due to changes in MOHS funding allocation (e.g., halting of remote allowances).

The lack of a national policy document describing how the Civil Service Code’s provisions specifically apply to MOHS staff in the current time period has prevented clear understanding and interpretation of policies at all levels, while reducing transparency. According to a district health official,

Many things are unclear to the [health facility] staff and even ourselves here at the district office. In Freetown they need to harmonize the Civil Code with actual policies to make official Ministry of Health policy guidelines. (DHMT staff, Outlying district)

**Fig. 3:**
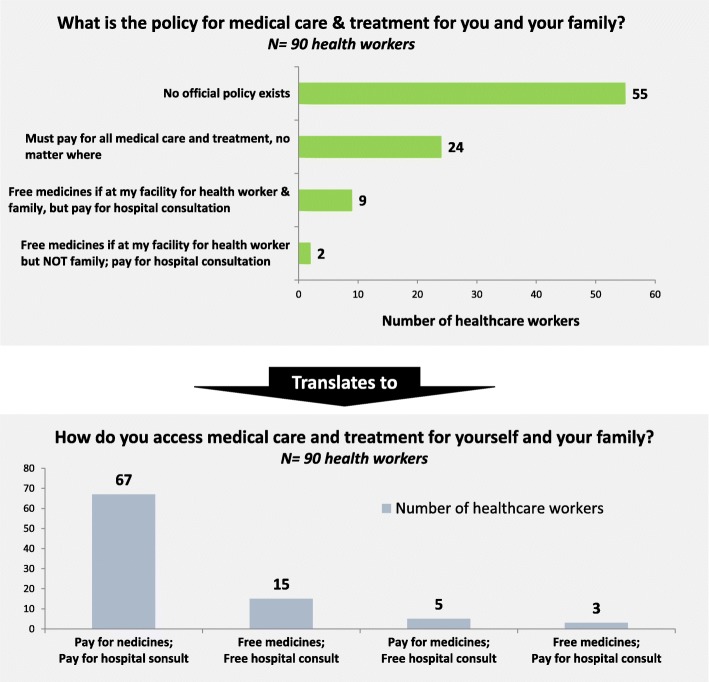
Policy knowledge versus policy implementation among health workers. Two graphs showing an example of policy ambiguity translated to inequitable policy implementation. Top graph shows health worker understanding of the policy regarding medical care provision for themselves and their families. Bottom graph shows how health workers actually accessed medical care

The policy ambiguity led to inconsistent and often inequitable implementation on the ground: some HCWs received benefits and opportunities while others did not. This is highlighted by the example of medical care coverage for HCWs and their families. Figure 3 shows the discrepancy between how HCWs interpreted the medical care policy and how they actually accessed medical care: some HCWs received free care and medications, others received either one or the other, while most paid out of pocket for all care and treatment. Often HCWs just did what their colleagues were doing or what they had heard from other people. Some asked the district or officials, but received conflicting information. One rural HCW explained:

To be honest, when me or my wife are sick, I use the medicines here [at the facility]. I do not know if that is recommended. No one at the district office knows when I ask, and sometimes I think it is [a] legal risk. (SECHN 17)

### Ineffective communication channels between government and health workers drive the knowledge and information gaps

Key information from the central government rarely reached HCWs, especially in outlying districts and remote areas. Communications from the national level about policy changes were ad hoc rather than part of an organized system where information is regularly conveyed between central government, districts, and HCWs. Further, there were no tracking mechanisms to ensure that the information reached its intended recipients. According to an official from an outlying district,

We find out new policies and things happening in the Ministry [of Health] sometimes from written memos they send, or when a colleague is in Freetown, or from our health staffs who got it from friends. Sometimes not at all. (DHMT staff, Outlying district)

There was also no systematic feedback channel for HCWs to voice their concerns and questions to the district and central government. As the HCW in-charge of a rural facility explained,

I carry to the district our challenges and things we want to know, but I don’t get the information. The [district] says they are trying and communicating to Freetown. But no response reaches to us. (CHA 50)

The absence of an effective two-way communication channel exacerbated the confusion among HCWs about the “study leave with pay” and “remote allowance” policies, and made it difficult for HCWs to rectify individual HR issues such as not being absorbed onto the national payroll.

Compounding the lack of regular communication channels was the absence of induction: new HCWs were never formally oriented to the MOHS’ policies and procedures before initial deployment. One rural HCW commented:

If only they convene all the staffs together in the district or Freetown and tell [us] all the policies in effect, we can know how to apply for scholarships or get our problems fixed like this broken [maternity] bed here. (CHO 57)

## Discussion

We found that many primary HCWs in rural Sierra Leone were dissatisfied with their jobs and intended to leave their post within 1 year. Consistent with a prior analysis in Sierra Leone [25] and global HRH retention literature, we found that job dissatisfaction was affected by inadequate living and working conditions, inconsistent financial remuneration, and poor support systems. However, we also identified a critical factor underlying these drivers of dissatisfaction—a lack of HCW knowledge of entitlements, policies, procedures, and national-level changes to HRH programming, coupled with lack of clarity in many of the policies. This directly translated to many HCWs not accessing their employee rights, not benefitting from advancement opportunities, and not being able to resolve individual HR challenges. Lack of proactive, regular communication of clear and accurate information from the government to HCWs—combined with the inability of HCWs to obtain updated and accurate information on their own—disempowered and frustrated them. It also fostered the sentiment among HCWs that the government does not care about the welfare of its employees.

To our knowledge, this is the first primary research investigating the HCW knowledge gap around HR policies and entitlements and its linkage to HCW job satisfaction and retention. If left unaddressed, this foundational problem threatens to undermine the effectiveness of other incentives and initiatives aimed at reducing rural HCW attrition in Sierra Leone. For example, the government may invest funds to provide additional career advancement scholarships for rural HCWs, but if the change in policy, criteria for candidate selection, and application procedures are not clearly communicated to HCWs, and if there are not effective avenues for HCWs to obtain clarification and information or follow-up on any application issues, the result may be confusion on the ground, perception of poor transparency, fractured implementation, and ultimately a muted effect on retention.

Based on our findings, we propose potential approaches to tackle the HCW knowledge and communication gaps in Sierra Leone, with the goal of improving HCW job satisfaction, distribution, retention, and productivity. These are tailored to Sierra Leone’s context, but may be applicable to other low- and middle-income countries (LMICs) experiencing similar HCW retention challenges.

**Fig. 4:**
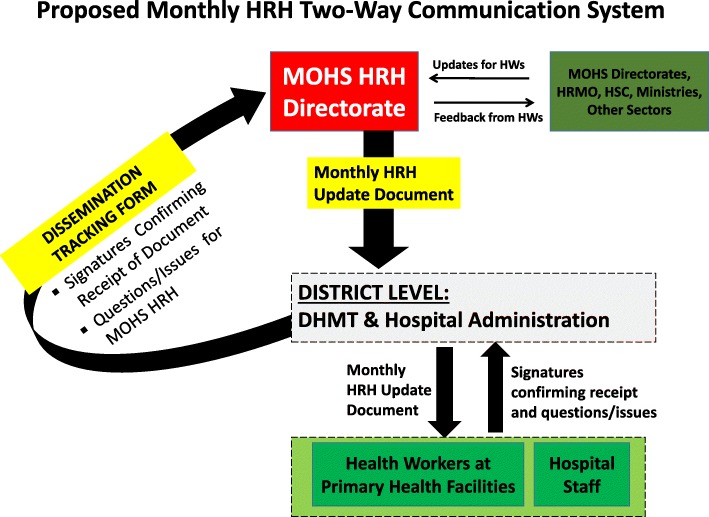
Potential two-way communication system between the Ministry of Health and health workers. Abbrev. MOHS = Ministry of Health and Sanitation, HRH = Human Resources for Health, DHMT = district health management team, HRMO = Human Resource Management Office, HSC = Health Service Commission

First, regular updates and communication could flow from the MOHS to the district health management teams (DHMTs) and health workers, with a feedback loop to enable central monitoring as well as receipt of health worker questions and concerns (Fig. 4). For example, the MOHS HRH Directorate could send a formal, monthly “HRH Update” document to each DHMT to provide to district staff and HCWs. The HCWs and DHMT could communicate back to the MOHS HRH Directorate raising issues and queries.

Second, an HRH Policies, Procedures, and Regulations document could be developed, which articulates in simple language how entitlements and policies in the generic Civil Service Code apply specifically to MOHS employees. The document could be regularly updated to reflect policy changes. This could reduce confusion among HCWs, ensure they follow the correct procedural steps, and provide guidance on what to do if a process has stalled. As an example of the type and format of information that may be useful for HCWs, we created a stepwise flow chart for the civil service absorption process based on our discussions with government agencies (see Additional file 3).

**Fig. 5:**
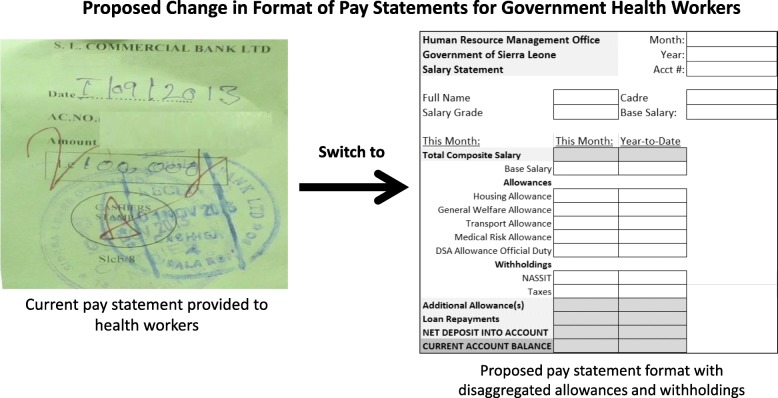
Potential change to health worker pay statement that disaggregates allowances and withholdings. Abbrev. DSA = daily subsistence allowance, NASSIT = National Social Security and Insurance Trust

Third, pay statements could provide a full breakdown of the “composite salary” (Fig. 5). This would include all allowances (housing, medical, transport) and all withholdings (social security, taxes), enabling HCWs to fully understand how they are being compensated.

Finally, induction training could be provided for all new entrants into Civil Service and for current HCWs, including explanation of their benefits and national policies. This would ensure that they possess the correct knowledge about all policies and procedures, rules and regulations, and entitlements.

## Conclusions

To improve rural workforce distribution and retention in Sierra Leone and similar country contexts, strong systems must be put in place for two-way communication between government and its HCWs; policies must be formulated with consensus and articulated without ambiguity; and HCWs must be provided with the necessary documentation about policies and procedures for accessing their rights. In Sierra Leone, the gaps in HCW knowledge and communication have been largely absent from discussions among government, donors, and NGO stakeholders about how to address health workforce challenges.

Global recommendations have been developed by the World Health Organization which serve as the framework for many countries as they consider how to address health workforce mal-distribution and attrition [31–33]. However, these recommendations do not address challenges in health worker knowledge and awareness of entitlements and policies, or the systems for how policies are articulated and communicated from the central government to its health workers. Although rural workforce retention challenges take different forms depending on the country and context, the structural gaps identified in this study likely exist in other LMICs. Our findings suggest that further research is needed in other LMICs to understand this challenge on a broader scale. Policymakers should consider addressing this driver of HCW dissatisfaction and its root causes in global and country-specific recommendations about increasing rural workforce retention. Failure to address and prioritize this key foundational barrier may limit the effectiveness of other initiatives undertaken to improve health workforce retention.

## Additional files

Additional file 1: Sierra Leone’s health system. Overview of Sierra Leone’s health system. Key background information about the country’s health system, including administrative districts and demographics, health facility levels, and cadres and staffing ratios. (PDF 472 kb)

Additional file 2: Card sort ranking method. Card sort method used to assess health worker priorities. Depiction and description of the card sort method used to measure health worker prioritization of factors affecting job satisfaction. (PDF 658 kb)

Additional file 3: Flowchart for absorption process. Flowchart showing the process for health worker absorption into Sierra Leone’s civil service*.* Flowchart showing the stepwise process for healthcare workers to be absorbed into the civil service and onto the government payroll. This was created by study investigators and is an example of the type and format of information that can more effectively inform healthcare workers about procedures to access their employee rights. (PDF 581 kb)

## Abbreviations

CHA: Community health assistant
CHO: Community health officer
DHMT: District health management team
HCW: Healthcare worker
HR: Human resources
HRH: Human Resources for Health
HRMO: Human Resource Management Office
LMIC: Low- and middle-income country
MCHA: Maternal child health aide
MOHS: Ministry of Health and Sanitation
SECHN: State-enrolled community health nurse
SRN: State-registered nurse
