# Supplementary material for: “If I had known, I would have applied”: poor communication, job dissatisfaction, and attrition of rural health workers in Sierra Leone
Source: Hum Resour Health. 2018 Sep 24;16:50. doi: 10.1186/s12960-018-0311-y (PMC6154815; doi:10.1186/s12960-018-0311-y)
Supplement: Supplementary file 1 — Sierra Leone’s health system. Overview of Sierra Leone’s health system. Key background information about the country’s health system, including administrative districts and demographics, health facility levels, and cadres and staffing ratios. (PDF 472 kb) [file 12960_2018_311_MOESM1_ESM.pdf]

Additional File 1: Overview of Sierra Leone's Health System

Administrative Districts and Demographics

The total population of Sierra Leone is an estimated 6,092,075 [1]. The gender distribution is 51% female and 49% male, with 50.4% of the population between the ages of 15-54 [2].

The country is divided into 13 administrative districts (Figure A). The capital Freetown, the biggest urban center, is located in the far west. Each district contains 13-15 chiefdoms, which represent the administrative subdivisions of the district.

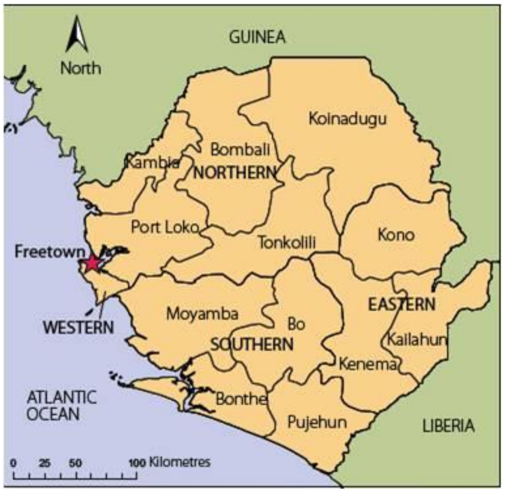

Figure A: **Map of Sierra Leone's Districts**

Health Facility Levels

Sierra Leone's public health system is comprised of five facility levels, as shown in Figure B. Health facilities are also classified as either a Hospital or a Peripheral Health Unit (PHU). According to 2017 data, Sierra Leone has 1,198 health facilities in total (21 hospitals and 1,177 PHUs) [3].

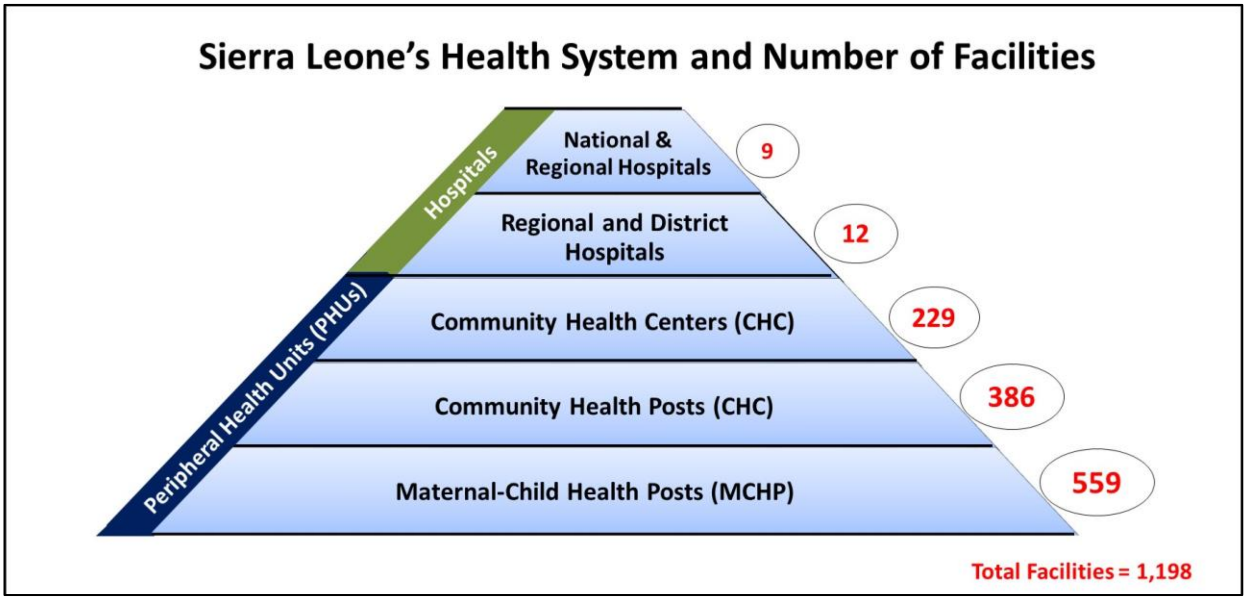

Figure B: **Sierra Leone's Health System**

The 3 national referral hospitals offering tertiary care are all located in Freetown, making it difficult for patients in outlying districts to access needed care for complex medical issues. Further, 12% of the entire national health workforce is concentrated at the 3 hospitals [4]. Each of the 12 upcountry districts has a District Hospital, which provides secondary healthcare for the district, as well as primary healthcare services for its immediate catchment. Each district has many PHUs which provide primary healthcare, thus serving as the entry point to the health system for the country's

majority rural population. In order of decreasing facility level, the PHUs consist of Community Health Centers (CHC), Community Health Posts (CHP), and Maternal Child Health Posts (MCHP). The PHUs refer patients up to the District Hospital.

Cadres of Healthcare Workers in Sierra Leone

There is a dire shortage of medical officers (doctors) in Sierra Leone, with only 0.3 medical officers per 10,000 population— the fourth worst density of medical officers in the world [5]. The limited number of doctors are largely concentrated in Freetown [4]. The lack of medical officers in rural areas reduces the level of services that can be provided outside of Freetown and the country's few upcountry cities, thus necessitating a high number of referrals to Freetown. It also increases the burden on the lower-level cadres working at the PHUs.

Primary healthcare is provided by Maternal-Child Health Aides (MCHA), State-Enrolled Community Health Nurses (SECHN), Midwives, Community Health Assistants (CHA), Community Health Officers (CHO), and State-Registered Nurses (SRN). SRNs only work at hospitals. A health facility is classified based on the services it provides, which in turn depends on the specific mix of cadres working at the facility. The below table shows the staffing ratios for each type of PHU [6].

**Staffing ratios at each level of PHU**	
**Facility Level**	**Cadres**
Community Health Center	2 CHO, 1 CHA, 2 SECHN, 4 MCHA, 2 Midwives
Community Health Post	1 CHA, 1 SECHN, 2 MCHA, 1 Midwife
Maternal-Child Health Post	3 MCHA

Community health workers (CHW) deliver primary healthcare services at community level (reproductive health, nutrition, communicable diseases, health promotion) and are managed by the in-charge of the nearest PHU. There are approximately 14,650 CHWs in Sierra Leone, according to 2016 data [3]. CHWs are not part of Sierra Leone's civil service. Many are financially supported by NGOs who utilize them in health programs. The level of training and role of CHWs varies widely depending on the district and the health activities they are engaged in.

The primary healthcare system is managed by the District Health Management Team (DHMT), which is led by the District Medical Officer (DMO) [6].

Healthcare services are free for breastfeeding and lactating women, as well as children under 5 years of age. All other patients must pay for medical services at government facilities, with the exception of treatment for malaria, tuberculosis, and HIV.

REFERENCES

1. United Nations Department of Economic and Social Affairs. (2013). World Population Prospects August 2013: 2012 Revision. UNDP; 2013. Accessed from http://esa.un.org/unpd/wpp/Excel-Data/population.htm.

2. Sierra Leone Ministry of Health and Sanitation (MoHS). Sierra Leone Service and Readiness Assessment (SARA). Government of Sierra Leone; 2011.

3. Sierra Leone Ministry of Health and Sanitation (MoHS). Human Resources for Health (HRH) Strategy 2017-2021. Government of Sierra Leone; 2017.

4. Sierra Leone Ministry of Health and Sanitation (MoHS). Human Resources for Health Country Profile. Government of Sierra Leone; 2017.

5. World Health Organization (WHO). Global Health Observatory: density of physicians by country. WHO; 2017. Accessed from http://www.who.int/gho/health_workforce/physicians_density/en/

6. Sierra Leone Ministry of Health and Sanitation (MoHS). Basic Package of Essential Health Services (BPEHS). Government of Sierra Leone; 2015
