# Supplementary material for: “If I had known, I would have applied”: poor communication, job dissatisfaction, and attrition of rural health workers in Sierra Leone
Source: Hum Resour Health. 2018 Sep 24;16:50. doi: 10.1186/s12960-018-0311-y (PMC6154815; doi:10.1186/s12960-018-0311-y)
Supplement: Supplementary file 2 — Card sort ranking method. Card sort method used to assess health worker priorities. Depiction and description of the card sort method used to measure health worker prioritization of factors affecting job satisfaction. (PDF 658 kb) [file 12960_2018_311_MOESM2_ESM.pdf]

Additional File 2
Card Sort Activity to Understand Healthcare Worker Priorities

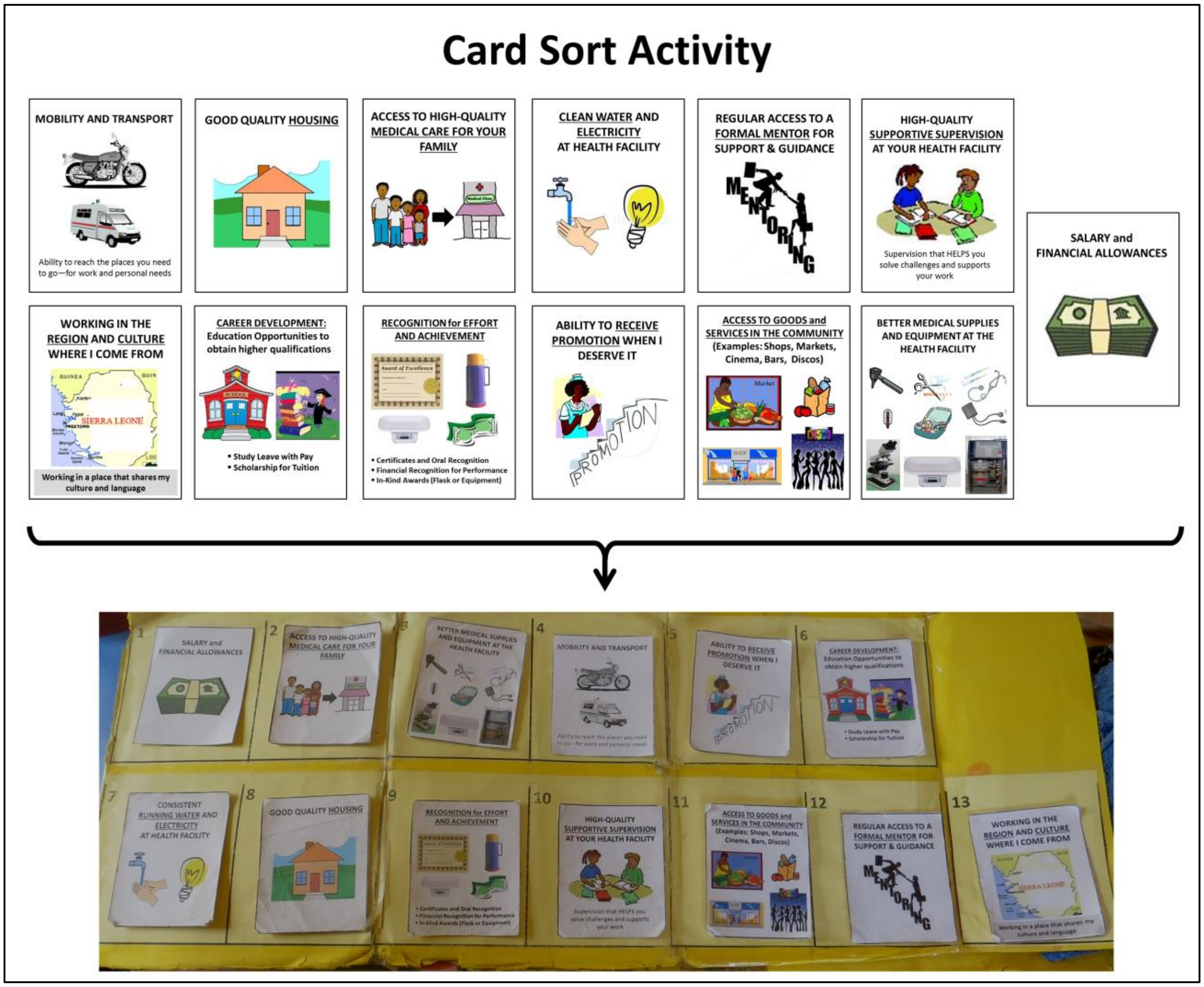

Description of the Method:

The healthcare worker was given 13 cards. Each card depicts pictorially a “factor that may affect a healthcare worker’s job satisfaction”. The 13 factors are shown in the image above. The healthcare worker was asked to place the cards in order from 1 to 13 based on which factors most affect his/her desire to stay or leave a health facility. After ordering the cards, the healthcare worker was asked to explain the rationale for their prioritization.

The rank-ordering was 'closed-ended': it did not allow for addition or removal of a card, and no ties were allowed. The closed-ended structure helped provide an understanding of *relative* prioritization because it required that the healthcare worker consider the importance of each factor in relation to the others, make definitive choices between factors, and articulate the rationale for his/her choices.
