# Supplementary material for: “If I had known, I would have applied”: poor communication, job dissatisfaction, and attrition of rural health workers in Sierra Leone
Source: Hum Resour Health. 2018 Sep 24;16:50. doi: 10.1186/s12960-018-0311-y (PMC6154815; doi:10.1186/s12960-018-0311-y)
Supplement: Supplementary file 3 — Flowchart for absorption process. Flowchart showing the process for health worker absorption into Sierra Leone’s civil service. Flowchart showing the stepwise process for healthcare workers to be absorbed into the civil service and onto the government payroll. This was created by study investigators and is an example of the type and format of information that can more effectively inform healthcare workers about procedures to access their employee rights. (PDF 581 kb) [file 12960_2018_311_MOESM3_ESM.pdf]

Process for Health Worker Absorption into Civil Service

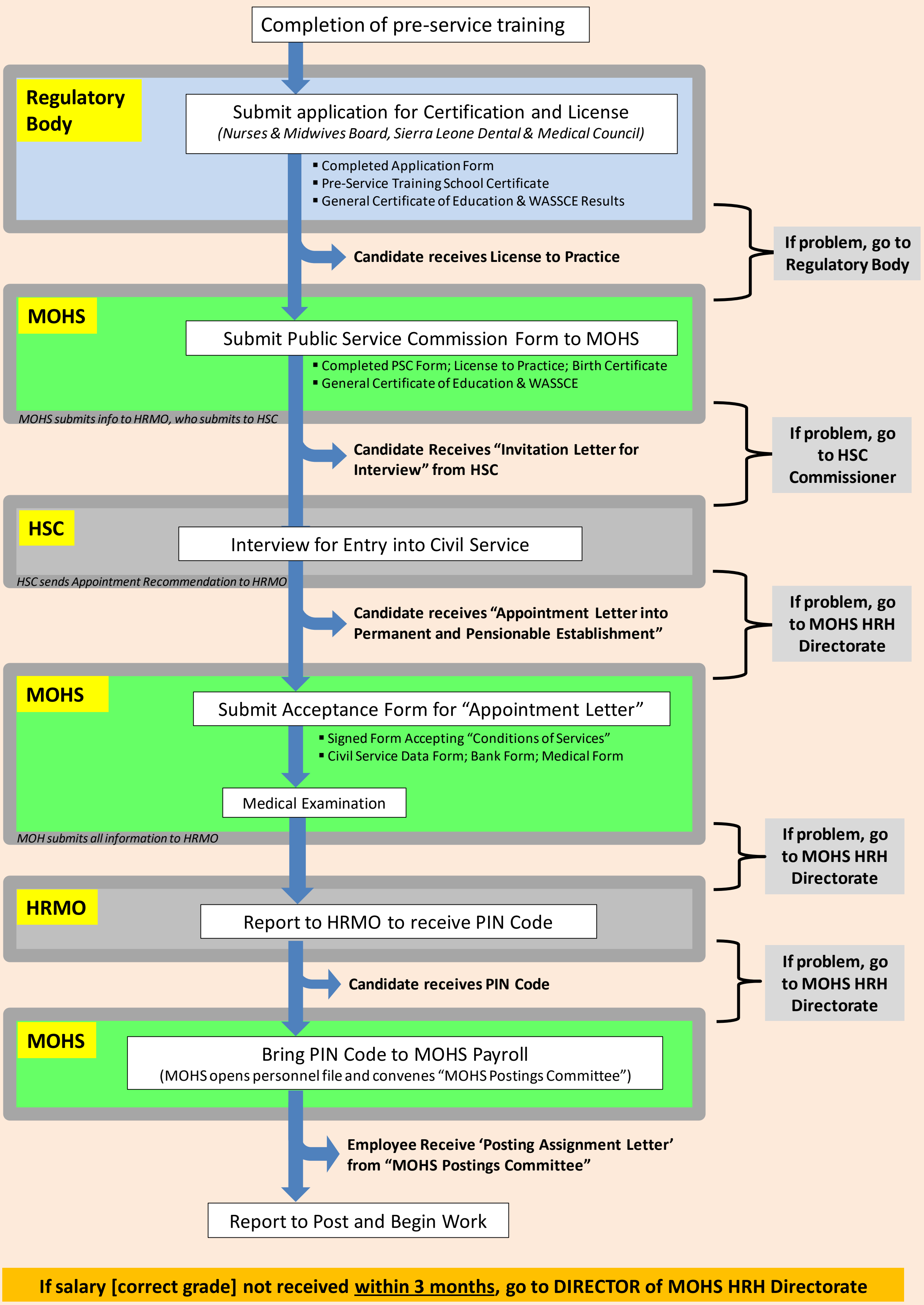

This flow chart was created by study investigators based on information from government staff
